# Specific structure and unique function define the hemicentin

**DOI:** 10.1186/2045-3701-3-27

**Published:** 2013-06-26

**Authors:** Xuehong Xu, MengMeng Xu, Xin Zhou, Odell B Jones, Edward Moharomd, Yuexin Pan, Guifang Yan, Donald D Anthony, Williams B Isaacs

**Affiliations:** 1 School of Life Sciences, Shaanxi Normal University, Xi’an, Shaanxi 710062, China; 2 Previous affiliation: School of Medicine, University of Maryland, Baltimore, MD 21201, USA; 3 Duke University Medical Center, Durham, NC, USA; 4 School of Medicine, University of North Carolina, Chapel Hill, NC, USA; 5 School of Public Health, Johns Hopkins University, Baltimore, MD, USA; 6 School of Medicine, Case Western Reserve University, Baltimore, MD, USA; 7 School of Medicine, Johns Hopkins University, Baltimore, MD, USA

**Keywords:** Extracellular matrix (ECM), Fibulin, Hemicentin, Embryogenesis, Tissue/Organ architecture, Cell division, Mitosis

## Abstract

Hemicentin has come a long way from when it was first identified in *C. elegans* as *him-4* (High incidence of males). The protein is now a recognized player in maintaining the architectural integrity of vertebrate tissues and organs. Highly conserved hemicentin sequences across species indicate this gene’s ancient evolutionary roots and functional importance. In mouse, hemicentin is liberally distributed on the cell surface of many cell types, including epithelial cells, endothelial cells of the eye, lung, and uterus, and trophectodermal cells of blastocyst. Recent discoveries have uncovered yet another vital purpose of hemicentin 1. The protein also serves a unique function in mitotic cytokinesis, during which this extracellular matrix protein plays a key role in cleavage furrow maturation. Though understanding of hemicentin function has improved through new discoveries, much about this protein remains mysterious.

## 

In the last two decades fibulins were rapidly recognized as a family of glycoproteins consisting of 6 or 8 members, fibulin-1, -2, -3, -4, -5, -7, and fibulin-6 and fibulin-8. Fibulin-6 and −8 are also referred to as hemicentin-1 and hemicentin-2, respectively [1]. Fibulins are defined as proteins consisting of a series of epidermal growth factor (EGF)-like modules, followed by a carboxyl-terminal fibulin-type module. Under this definition, 5 proteins (fibulin-1, -2, -3, -4, -5) were traditionally categorized into this family with the more recent addition of fibulin-7 [2]. Hemicentin-1(hem-1/fibl-6) and hemicentin-2 (fibl-8) were qualified for this family as well [1,3]. However, recent research identifying a function unique to hemicentin and a novel domain at its amino acid terminal have distinguished hemicentin from the fibulin family.

Hemicentin was first named in *C. elegans* as him-4 (short for High incidence of males) and is aptly one of genes responsible for increased X chromosome loss in nematodes [4-6]. Two orthologs discovered in vertebrate animals were subsequently termed hemicentin-1 and hemincentin-2. These molecules are characterized by a vWA (von Willebrand/Integrin A) domain attached to the amino acid-terminal of the signal peptide and hemicentin motif (hem motif) followed by approximately fifty Ig (immunoglobulin) modules. The vWA domain and Ig module together take up > 80% of the molecular structure and are responsible for predicting the function of hem on hemidesmosomes [6]. Thus, although hemicentin fit the criteria for fibulin, structural differences from the rest of the fibulin family members support treating them as an independent protein family. Recent findings on hemicentin’s unique function in the cell cycle have served to strengthen this fact [7-11].

When a 90-kDa calcium-binding secreted glycoprotein was termed the first fibulin, this extracellular matrix (ECM) protein was known to function within fibrillar basement membrane and independently as a BM-90 [12-14]. The second family member, fibulin-2, was identified shortly thereafter [15]. Then S1-5/EFEMP1, BMP1/EFEMP2/H411, DANCE/EVEC/UP50 and TM-14 were also merged into the fibulin family as fibulin-3, -4, -5 and −7, respectively [2,3,16-18]. These proteins were arranged in the fibulin family by categorizing the secondary structures of fibulin-type and EGF-like modules found at their carboxy-terminals.

To date, the hemicentin family is composed of three members (Figure 1). The two domains following a signal peptide at the amino acid terminal, vWA domain and a chain of Ig modules, are the key characteristics of every family member. Though, very little is known about the function of these two modules, they are signature structures found in every hemicentin gene. These three isoforms take up a significant percentage of the entire protein sequence. In *C. elegans*, this signature portion contains4801 amino acids, more than 90%of the full hemicentin molecule. In mouse, hemicentin-1 and -2’s signature portions are 4473 and 4389 amino acids long, respectively, and account for 80% of each gene. Table 1 summarizes the genetic composition of hemicentin in each animal (Table 1).

**Figure 1:**
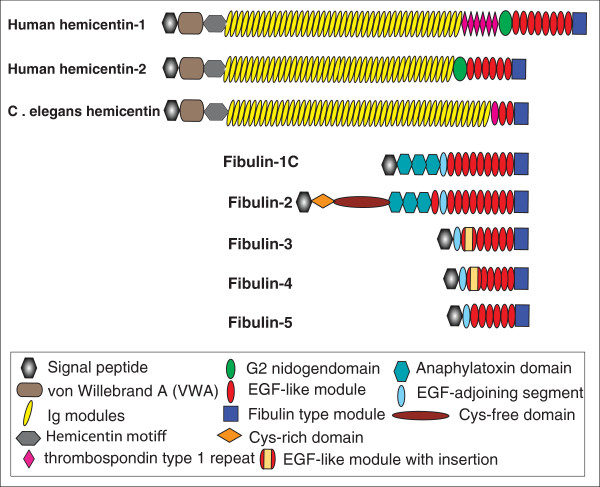
**Schematics comparing the structures of fibulins and hemicentin family members in both *C. elegans *and human.** The von Willebrand A domain (vWA), located behind the amino acid terminal signal peptide and the long chain of immunoglobulin modules, which follows the hemicentin domain, encompasses over 80 percent of the entire translational length of hemicentin genes (figure not to scale).

**Table 1:** Hemicentin nomenclature in model animals

**Animal model**	**Hemicentin nomenclature**	**Gene symbol**	**Exon**	**Location**	Transcripts (bp)^*^	**Translation length (AA)**	**vWA and Ig**	
							**Length (AA)**	**Percentage (%)**
*C. elegans*	Hemicentin Fibulin6 Him-4	*Him-4*	*62*	Chromosome X: 9,717,568-9,753,706	15723/15792	5175/5198**	4801	92.3624
*Danio rerio*	Hemicentin 1	*HMCN1 ZFIN*	107	Chromosome 20: 34,182,641-34,317,215	17173/17450	5616/5616**	4458	79.380
	Hemicentin 2	*HMCN2*	95	Chromosome 8: 33,554,295	14739	4913	-	-
*Xenopus tropicalis*	Hemicentin 1	*HMCN1*	106/115	Scaffold GL172705.1: 1,652,334-1,775,760	15279/16818	5092**/5605	4359	85.6049
	Hemicentin 2	*HMCN2*	98	Scaffold GL172827.1: 1,836,154-1,900,680	14919	4972	3873	77.8962
*Mus musculus*	Hemicentin 1 Fibulin6	*HMCN1*	107	Chromosome 1: 152,410,657-152,840,181	16905/16554	5634^**^/5517	4473	79.393
	Hemicentin 2 Fibulin8	*HMCN2*	98	Chromosome 2: 31,169,935-31,316,258	15646	5100	4389	86.0588
*Homo sapiens*	Hemicentin1 Fibulin6	*HMCN1*	107/106	Chromosome 1: 185,703,683-186,160,081	18208/17861	5635**/5518	4479	79.4854
	Hemicentin2 Fibulin8	*HMCN2*	92	Chromosome 9: 133,028,269-133,309,510	16098	5366	-	-

Notes: * If the gene has more than two transcripts, the two largest are listed.

** Calculation on number of amino acids between the vWA domain and the last Ig module is based on this translation length.

During evolution, two vertebrate orthologs (hem-1 and hem-2) developed from the single *C. elegans* hemicentin. Comparison between these two genes exhibits some interesting similarity in their molecular structures. Alignment between hemicentin family members reveals an amino acid gap between the vWA domain and the first Ig module (226 aa in Ce hemicentin, 222 in hem-1 and hem-2), which have multiple conserved amino acid sequences (Figure 2). Two motifs, AVKQKKVHLM and IPVDKHLSELTISLSGD, at the N-terminal in *C. elegans* hem developed into identical motifs, A(V/I)QASKVHLS and IPFDPSLKEVT(V/I)SLSGP, with Valine in hem-1 and Isoleucine in hem-2. In the middle of the gap, a LKHTIRVFG *C. elegans* hem developed into either GRHSVRITG or GRHSVRMTG in vertebrate hem-1, GRHTVRITG in *X. tropicalis*hem-2, or GRHSVRISG in mouse hem-2. At the carboxyl terminal of this domain, the motif shows more diversity between two isoforms, FFLKVTGYD or FFIKIIGYD encoded on one region of in hem-1 and FFMKVNGTD or FYLKVKGKD encoded in another region. Between motifs II and III, III and IV, and after IV, amino acid sequences in hem-1 and hem-2 no longer correspond, but do display homogeneity across species.

**Figure 2:**
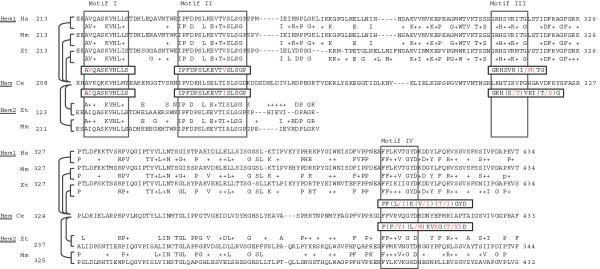
**Similarity analysis on hemicentin family members shows highly conserved amino acid regions between the amino acid terminal vWA domain and the first Ig module.** Comparative alignment between *C. elegans* (Ce) hemicentin, and hemicentin 1/hemicentin 2 exhibits four specific sequence motifs shared by corresponding genes in human (Hs), mouse (Mm), and Xenopus (Xt). These conserved sequences distinguish hemicentin family members across multiple species.

Previous review articles based on hereditary disease studies and basic research results gathered from animal models, demonstrated the various functions of fibulins in human disorders [3,18-20]. Recent progresses discerning the function of hemicentin in various animal models have drawn increasing attention.

Histological and histochemical analyses in *C. elegans*, mouse, and zebra fish models suggest hemicentin functions as an extracellular adhesive, forming cell-cell and cell-basement membrane adhesion that hold cells together and maintain tissue and organ integrity [6,7,21]. In *C. elegans,* hemicentin forms linear structures between somatic cells to anchor the epidermis to the uterus, the mechanosensory neurons, and the intestine. The protein also assembles an elastic, fiber-like structure which surrounds the nematode body-wall muscles [6,22]. In mouse, hemicentin assembles into closed sheets that are insinuated between cells. This structure completely surrounds cells in certain tissues, such as dermal epithelial cells, stratified corneal epithelial cells, and tongue epithelial cells [22]. Hemicentin is also distributed across the entire inside surface of the lens. In the retina, hemicentin assembles on the pigmented retina epithelium and choroid basement membranes to form cell-ECM-cell “sandwiches” that flank collagen XVIII in Bruch’s membrane [23]. Mutations in hem-1 have been linked to age related macular degeneration (ARMD), indicating the importance of this protein in retinal function [8] and providing a new avenue for understanding ARMD, the main cause of blindness in the western world.

In mouse embryonic development, hemicentin is co-localized with desmosomal cadherin desmocollin-3 on the periphery of blastocytic trophectoderm cells originating from the first differentiation after fertilization and oocyte formation. This peripheral distribution is observed as a punctuated linear structure during morula stage. Before this stage, the proteins are first observed on the embryonic cell surface of four to six cell stages, when each cell is still totipotent. The protein’s distribution in cells of earlier embryonic stages remains unclear [23]. Interestingly, genetic analyses on four-cell stage mouse embryo have recently unveiled a distinctive function of hemicentin in mitotic cytokinesis [7].

As previously described, extracellular matrix proteins all function in cell-cell anchoring and are vital for maintaining cell adherence to tissue and organ basement membrane. In addition to this well-known role as an ECM, hemicentin-1 has recently been discovered to have another unique function [7]. Recent evidence has uncovered a vital role hemicentin plays in normal embryonic development. Before its distinctive peripheral distribution and architectural role in the fully developed mouse, hem-1 plays a critical role in proper mitotic cytokinesis. In wild type mouse embryo, after male and female nuclei fusion but prior to cytokinesis, extracellular hemicentin proteins previously distributed evenly on the cell surface, relocate to the eventual cleavage furrow location. Hemicentin condense on the furrow, co-localized with intracellular myosin IIB and actin to form a contractile ring complex with hemicentin studding the membrane surface and anchoring the cytoplasmic ring composed of tightly woven myosin/actin molecules (Figure 3). However, cell membrane receptors integrating the ECM hemicentin with intracellular myosin/actin have yet to be discovered.

**Figure 3:**
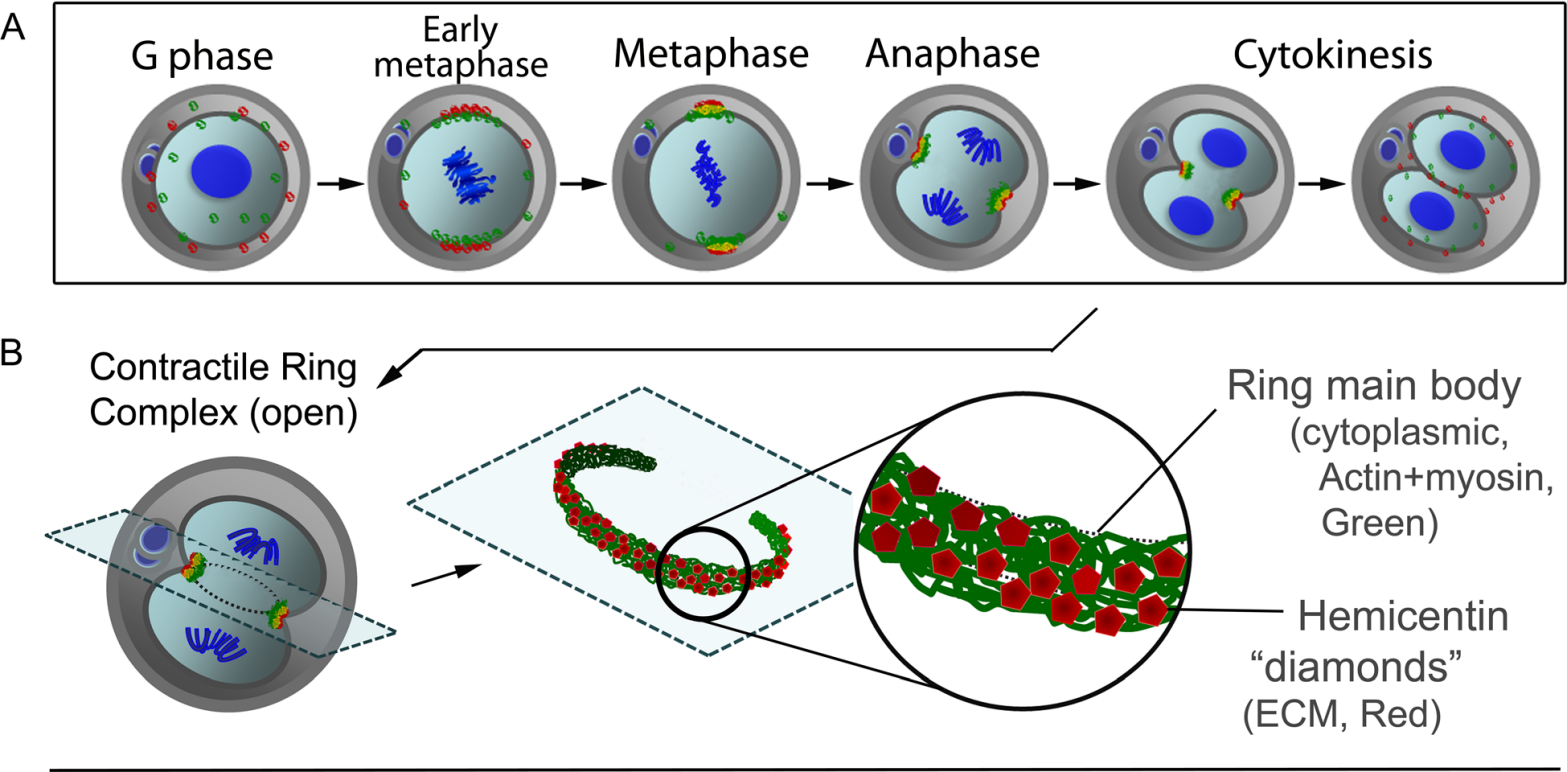
**Schematic representation of hemicentin (Red) and non-muscle myosin IIB / actin (Green) predicted migration during mitosis. (A)** Extracellular hemicentin and intracellular myosin this & actin localization during contractile ring complex formation—from G-phase to metaphase and extending into cytokinesis. During G-phase, cytoplasmic myosin and actin begin congregating on the internal cell surface to form the contractile ring. Simultaneously, the extracellular, membrane bound hemicentin travel from their previously homogeneous distribution to localize in the cleavage furrow. By metaphase, this process results in an open ring composed of a solid weave of intracellular myosin and actin held in place by hemicentin studding the band extracellularly. This contractile ring then shrinks during cytokinesis with hemicentin serving as anchor points of contraction. When cell division is complete, all molecules return once again to their previous even distribution. **(B)** Artistic 3 dimensional rendering of the dynamic process describing an open ring contracting over time.

In hemicentin-1 deficient mice, embryonic cells cannot complete mitotic cytokinesis to form daughter cells. These cells can, however, complete the preceding elements of mitosis. Thus, the majority of mutant blastomeres arrest at the one-cell or two-cell stage with multiple nuclei, indicating the number of attempted mitosis and incomplete cytokinetic cleavage furrow retractions [7]. The loss of hemicentin leading to multinucleate cells was previously observed in *C. elegans* germ lines and was concluded to be the result of “occasional fusion of neighboring cells” [6]. However, recent studies have shown that the multinucleate cells found in mouse pre-implantation blastomeres are caused by hemicentin-1 defects, and suggesting that the previously disclaimed observations in *C. elegans* may be due to hem-1 defects as well*.* The study in mouse used three different methods to delete HMCN1, homogenous recombination, RNAi HMCN1 knockdown, and induced parthenogenesis on HMCN1^+/−^, which all resulted in arrested blastomeres with multiple nuclei. The genetic manipulations and resultant multinuclear blastomeric findings indicate that the “fused cells” found in *C. elegans* are actually the result of HMCN1 defects introduced in the study [7]. Recently, zebrafish hem-1/fibl-6 and hem-2/fibl-8 were reported to be highly connected to epidermal-dermal development in relation to adjacent basement membrane [21,24]. HMCN1 has been proven to be one of the genes related to fin basement malformation, characterized as Fraser syndrome [21]. Double knock-down defects of HMCN2 and FBLN1 (but not HMCN2 alone) proved that both genes are crucial for epidermal–dermal junction formation and fin mesenchymal cell migration during zebrafish development [24]. The diverse functions of the hemicentin on various developmental stages of both invertebrates and vertebrates from cell division to tissue/organ architecture indicate conserved functioning of the ancient genes.

Loss of hemicentin-1 causes acytokinetic cell divisions in mouse early development, a recently discovered novel function for this extracellular matrix protein. This function distinguishes hemicentin from other ECM families, including the fibulin family to which it was previously categorized. In addition to calling into question protein nomenclature, this finding evokes many new questions on the function of this ECM protein in cell cycle. What is the membrane receptor(s) which link hemicentin to the internal cytoskeletal structure? What are differences in hemicentin found on the contractile ring complex at mitotic status and G0 stage? Does hemicentin play a role in promoting cell differentiation and tissue architecture? If so, what are their receptors or trans-membrane protein players? Many questions regarding the hemicentin protein family remain unresolved and waiting for investigation.

## Ethical approval

In this review, no any experimental research carried out on humans or human tissues /cells.

## Competing interests

The authors declare that they have no competing interests.

## Authors’ contribution

XHX conceived the study, participated in its design, and drafted the manuscript. MMX, YP and GY carried out the similarity analysis on hemicentin family members and representative diagram of mitosis. XZ, EM and OBJ performed the nomenclature analysis. DDA and WBI drafted and edited the manuscript. All authors read and approved the final manuscript.
